# Resource Misallocation and Energy-Related Pollution

**DOI:** 10.3390/ijerph18105158

**Published:** 2021-05-13

**Authors:** Ling-Yun He, Xiao-Feng Qi

**Affiliations:** College of Economics, Jinan University, Guangzhou 510632, China; qxfeng1994@126.com

**Keywords:** resource misallocation, environmental pollution, energy

## Abstract

Developing countries face the conflict between economic development and environmental protection. Resource misallocation will not only affect the effectiveness of economic development, but also have environmental impacts. Based on two large-scale enterprise databases in China, this paper measured the level of enterprise resource allocation, and further used empirical research methods to investigate the environmental impact of enterprise resource misallocation and specific mechanisms. The results show that the low efficiency of resource allocation will harm the quality of China’s environment. Further investigation, resource misallocation is accompanied by an increase in total energy input, a decrease in the labor-to-energy ratio and the capital-to-energy ratio, and a loss of energy efficiency, which in turn affects the environmental performance of enterprises. China is the largest developing country in the world, and research on China’s environmental and economic issues is important. The conclusions of this paper can provide experience and suggestions for other developing countries to improve environmental quality and promote sustainable development from the perspective of resource misallocation.

## 1. Introduction

In the process of China’s rapid development, the extensive development mode of high energy consumption, high pollution and high emission has brought huge environmental loss and energy consumption, and there is a conflict between economic progress and environmental protection. Since 2011, China has become the largest industrial country in the world, where many pollutant emissions are among the highest in the world and have exceeded environmental tolerance. In 2018, only 121 of China’s 338 prefecture-level cities met ambient air quality standards, accounting for only 35.8% (Data comes from “2018 Bulletin of China’s Ecological Environment”.). Serious environmental problems not only restrict the quality of China’s economic development, but also endanger the health of residents. President Xi Jinping has repeatedly emphasized that lucid waters and lush mountains are invaluable assets. This requires that we cannot separate economic development from ecological protection, nor can we follow the old path of pollution first and then governance. The core of implementing the development concept of the “Two Mountains Theory” lies in correcting and solving the potential factors that simultaneously restrict economic development and environmental protection. Economy and environment are closely linked. The essence of sustainable development in China and other developing countries is to find a new balance between environment and economic development.

Existing studies believe that resource allocation has become an important factor affecting the economic development of countries [1,2], and it is generally believed that long-term resource misallocation will lead to the loss of total factor productivity (TFP) [1,3,4,5]. While resource misallocation has an impact on economic development, the process of misallocation may be accompanied by the loss of environmental quality. At the macro level, resource misallocation has a lock-in effect on the production mode of energy-intensive industries; the transformation of industrial structure is difficult, as enterprises are overly rely on tangible factors, which leads to the lack of independent innovation motivation [6]; and inter-regional division is less efficient. At the micro level, resource misallocation can lead to the inefficiency of energy input, form entry barriers for potentially high-efficiency green enterprises, raise the exit barriers for backward production capacity and low-efficiency enterprises; and make high-energy-consuming enterprises have path dependence. All these will bring about the loss of environmental quality. Therefore, the improvement of environmental quality and economic development must pay attention to the impact of resource misallocation.

Resource allocation not only affects a country’s economic development, but also affects environmental quality. Long-term resource misallocation will restrict economic growth and deteriorate environmental quality. However, from theoretical and existing literature research, how resource allocation affects a country’s environmental quality is rarely addressed. In addition, the existing research on China’s environmental impact is mostly at the macro level, lacking evidence support at the micro level. Focusing on China’s environmental issues from the perspective of resource misallocation and clarifying the mechanism by which resource misallocation affects enterprise pollution emissions are of great significance for China and other developing countries to achieve green and sustainable development.

This paper may make incremental contributions from the following points: (1) Resource misallocation not only affects economic development, but also affects environmental quality. Most of the existing literature focuses on the economic impact of resource misallocation [1,2,5], the causes of resource misallocation [7,8,9,10,11] and whether policies [12,13] or other factors [14] have exacerbated resource misallocation, while few studies have investigated the environmental impact of resource misallocation. This paper incorporates environmental issues into the research framework, and provides a new perspective for the improvement of China’s environmental issues. (2) Although a small number of studies have indirectly mentioned the environmental impact of resource misallocation under other research topics [6,15,16], none of them have discussed resource misallocation itself and the specific mechanism in detail, and the aforementioned works were all conducted at the regional or industrial level, lacking evidence at the enterprise level. This paper uses enterprise data to demonstrate and analyze the environmental impact of resource misallocation for the first time, and examines the specific mechanism of resource allocation affecting pollution emissions, which complements the shortcomings of the research in the micro-field. (3) Many developing countries have the problem of resource mismatch (e.g., India [1] and Ukraine [17,18]), and they are also faced with the dilemma that it is difficult to balance economic development and environmental protection. As the largest developing country in the world, this paper takes China as the research object for an in-depth discussion. The conclusions and policy enlightenments can provide experience and reference for other developing countries to reduce misallocation, improve the environment, and achieve sustainable development.

The rest of the paper proceeds as follows. Section 2 is the literature review and theoretical analysis. Section 3 is the introduction of database and index measurement. In Section 4, we describe our empirical strategy, and then present the main empirical results of the research hypotheses. Section 5 is heterogeneity analysis. The conclusions and policy implications are in Section 6.

## 2. Literature Review and Theoretical Hypotheses

### 2.1. Literature Review

Traditional economic growth theory believes that TFP is the only factor that causes the income gap between countries [19]. As the process of world economic integration accelerates, traditional economic growth theories cannot fully account for this income gap between countries. In this background, resource misallocation has become a new perspective to study productivity differences between countries [2,20]. This section focuses on the definition of resource misallocation as well as the causes of resource misallocation, its measurement methods and economic and environmental impacts, providing a literature foundation for our study.

#### 2.1.1. Causes and Measurement Methods of Resource Misallocation

In the perfectly competitive market structure, resources will flow spontaneously from the low rate of return sector to the high until the marginal returns of each sector are equal. This economic basis has become the criterion for judging whether there is resource misallocation. According to the differences of specific research objects and contents, the existing literature holds that financial market distortion [7,21], local protection, imperfect market structure [8], incomplete information [10], and unreasonable industrial policies [9] and so on may cause resource misallocation. In economic activities, resource misallocation is generally not caused by a single factor, but the result of the interaction of multiple factors.

Olley and Pakes [22] think that under the condition of competition, the relationship between enterprise size and productivity can reflect the efficiency of resource allocation. With the gradual improvement of micro data and the introduction of heterogeneous enterprise theory, Hsieh and Klenow [1] constructed an analysis framework from the enterprise to macro economy levels based on Melitz [23], which had a profound impact on the measurement methods of resource misallocation and the relationship between resource misallocation and TFP. Aoki [24] improved the study of Hsieh and Klenow [1] by abandoning the hypothesis of the production function, providing a basis for the international comparison of resource misallocation. In addition, some studies have measured resource misallocation by measuring the discreteness of TFP [25], which is quite simple and intuitive, but ignores the heterogeneity at the enterprise level.

Through the analysis of different resource misallocation measurement methods, it can be found that the framework of Hsieh and Klenow [1] has unique advantages in the analysis of resource misallocation of enterprises. In view of the fact that this paper mainly studies the environmental impact of China’s resource misallocation from the perspective of enterprises, the study of Hsieh and Klenow [1] has naturally become one of the main methods of this paper.

#### 2.1.2. Economic Impact of Resource Misallocation

On the economic impact of resource misallocation, TFP or economic growth is still the focus of attention. Hsieh and Klenow [1] studied the impact of resource misallocation on TFP in China and India. Their research found that China’s monopoly system and India’s licensing system have resulted in different levels of resource misallocation. If the resource allocation of the two countries reaches the level of the United States, the manufacturing TFP will increase by 40% and 50%, respectively. Based on the research of Hsieh and Klenow [1], Restuccia [5] argued that severe inefficiency in resource allocation can explain the “middle income trap” phenomenon in Latin America to a certain extent. The research of Brandt et al. [2] found that the misallocation of resources between the state-owned sector and the non-state sector was the main cause of resource misallocation in China, where the inter-provincial and cross-sector mismatch of labor and capital resulted in a 20% loss in total TFP, further supporting the conclusion of Hsieh and Klenow [1].

The difference in resource allocation provides a new perspective for explaining the difference in the level of economic development among countries. However, only paying attention to the speed of economic development can easily overlook the excessive energy and environmental consumption, which leads to inefficient and unsustainable economic development. Therefore, while paying attention to the impact of resource misallocation on economic development, it is important to clarify its possible environmental impact. This is also an important starting point for this paper.

#### 2.1.3. Environmental Impact of Resource Misallocation

There is no doubt that while the resource misallocation has an impact on the economy, it will also affect the environment. Based on the provincial panel data of China from 2002 to 2015, Bian et al. [6] found that market segmentation restricted the free flow of factors among regions, resulting in the deterioration of regional resource allocation and the increase in environmental pollution. However, in the empirical research, it only regards resource misallocation as the transmission mechanism of market segmentation affecting regional environmental quality, and does not explain the impact of resource misallocation on environmental quality and the possible impact path in theory and empirical research. Wang et al. [15] found that environmental regulation has a U-shaped effect on the improvement of regional resource misallocation. Under certain conditions, environmental regulation can alleviate resource misallocation and improve ecological efficiency. However, this study has not paid attention to the mechanism of resource misallocation on ecological efficiency. Lin and Du [26] found that factor market distortion restricts the improvement of China’s energy efficiency, and the correcting factor market distortion can increase China’s energy efficiency by about 10% and reduce energy waste by 14,500 tons of standard coal. The excessive consumption of energy is the main source of pollution in economic activities, but the study did not directly pay attention to the impact of factor distortion on pollution emissions, only showing that the improvement of energy efficiency is an effective way to save energy and reduce emissions in China.

Sustainable development requires that we cannot look at the economy and the environment separately. By reviewing the literature, we can find that the existing literature pays more attention to the economic impact of resource misallocation. Resource misallocation will undoubtedly have an adverse impact on the environment through the inefficient use of resources. Unfortunately, the existing literature does not pay sufficient attention to the environmental effects of resource misallocation. In addition, limited by the availability of enterprise data, the existing literature studies on resource misallocation and the environment are mostly at the macro level, ignoring heterogeneity at the enterprise level. Therefore, from the micro level, this paper focused on the environmental performance of enterprises from the perspective of resource misallocation, and deeply investigated the mechanism of resource misallocation affecting environmental performance, which provides micro evidence for resource misallocation and environmental research, and also provides a new perspective for China and other developing countries to achieve the sustainable development of the economy and the environment.

### 2.2. Theoretical Hypotheses

In this subsection, we mainly put forward the research hypotheses of this paper. The research hypotheses in this subsection will lay the foundation for the empirical analysis.

Firstly, according to the literature review of the economic and environmental impacts of resource misallocation, we can see that resource misallocation not only has an economic impact, but also has adverse impact on environmental quality. Therefore, based on the literature review, we propose the first research hypothesis:

**Hypothesis** **1.** *Resource misallocation not only affects economic performance, but also affects environmental performance by increasing the pollution emissions of enterprises.*

Secondly, energy is a major factor affecting economic development and environmental quality. It is generally believed that the inefficiency of energy input is an important cause of environmental problems. From 1997 to 2016, China’s total energy consumption grew at an average rate of 6.33% (According to the data of the “China Statistical Yearbook”, which is calculated by the authors.). According to the “World Energy Statistics Yearbook 2017”, China accounted for approximately 23% of global energy consumption in 2016, while China’s gross domestic product (GDP) in 2016 accounted for approximately 14.8% (Data comes from “International Statistical Yearbook, 2017”) of the total global economy. To achieve the coordinated development of economy and environment, it is urgent to change the extensive development mode relying on factor input and realize the intensive utilization of energy factors. The misallocation of resources in enterprises makes it impossible to achieve an optimal allocation of capital and labor factors, which is inevitably accompanied by the inefficiency of energy input. This may also be an important reason for the inconsistency between China’s total energy consumption and the economic development achievements (GDP) in the world. Therefore, we propose the second research hypothesis:

**Hypothesis** **2.** *Resource misallocation affects the environmental performance of enterprises by influencing the use of energy factors. Specifically, resource misallocation leads to an increase in the average energy input, resulting in double losses of economic efficiency and environmental performance, which are not conducive to the coordinated development of China’s economy and environment.*

Thirdly, according to the firm theory in microeconomics, there are different degrees of substitution and complementarity among the factors in the production process. Therefore, the resource misallocation measured by capital misallocation and labor misallocation will also affect the optimal factor input ratio. When the allocation of capital and labor is excessive or insufficient, the enterprise will deviate from the optimal factor input ratio. The improvement of environmental quality requires the optimization of factor allocation structure, so that resources can be allocated to high-efficiency enterprises, thereby improving the input–output efficiency and reduce environmental losses. The resource misallocation is just a reflection of the deviation from the requirement of sustainable development. On the basis of comprehensive analysis, this paper holds that the resource misallocation may cause enterprises to deviate from the optimal allocation structure, which is not conducive to the coordinated development of China’s economy and environment. Therefore, we propose the third research hypothesis:

**Hypothesis** **3.** *Resource misallocation affects the factor allocation structure in the production process, and thus affects the environmental performance of the enterprise. Specifically, resource misallocation may cause enterprises to deviate from the optimal labor–energy ratio and capital-energy ratio, which is not conducive to the improvement of environmental quality.*

Finally, China’s total energy consumption and energy consumption structure make it difficult for economic growth to decouple from pollution emissions. The improvement of energy efficiency is an important starting point for alleviating environmental and climate change problems. How to improve the efficiency of energy input has become an important aspect of balancing economic development and environmental protection. Based on the purchasing power parity method (PPP), China’s energy consumption intensity (the amount of energy consumed per unit of GDP) is approximately 1.38 (Data comes from “International Statistical Yearbook, 2017”.) times that of the world average. The 2016 Energy Efficiency Market report released by the International Energy Agency (IEA) also points out that China is a major contributor to the global energy efficiency improvement. The uneven energy efficiency of enterprises, the large scale and number of enterprises with low energy efficiency and the production preference for using more energy may be the reasons for China’s low energy efficiency [27]. Resource misallocation may further worsen the above situation, which is not conducive to the improvement of energy efficiency. We believe that the optimal allocation and dynamic adjustment of resources can be achieved through reasonable mergers and reorganizations among enterprises, thereby improving energy efficiency as a whole. Therefore, we propose the fourth research hypothesis:

**Hypothesis** **4.** *The improvement of resource misallocation is conducive to improving energy efficiency and mitigating negative environmental externalities in the process of energy consumption, which can help to realize the improvement of environmental quality and economic development.*

As the largest developing country and the second largest economy in the world, China’s economic and environmental issues are important. The existing literature has fully demonstrated the economic effects of resource misallocation, but there is little research on its environmental impacts. Will resource misallocation have an impact on the environment? In what ways does it affect the environment? Clarifying the environmental effects of resource misallocation may be an important breakthrough for China and other developing countries to improve environmental quality. The following part of this paper will verify the above four research hypotheses by building an empirical model.

## 3. Data Description and Index Measurement

This section mainly introduces the data in this paper and the measurement methods of relevant indicators, providing data support for empirical research.

### 3.1. Data Introduction

The first enterprise database used in this paper was the Annual Survey of Industrial Firms Database (ASIF). The database, established by China’s National Bureau of Statistics, includes all state-owned enterprises in China and non-state-owned enterprises above the scale (the main business is CNY 5 million and above). In addition to the economic census data, these data currently constitute the largest enterprise-level database available. In the database, manufacturing enterprises account for more than 90%. Industrial activities are the main source of pollution, so the database is very representative as the basic database to investigate the environmental effects of resource misallocation. ASIF has a large sample size and a wide range of coverage, however, there are also abnormal indicators. We removed outliers through specific indicators (see Appendix A).

The China industrial enterprise database mainly includes the indicators of input, output and financial status, but does not include the non-expected output indicators in the production process (mainly referring to the relevant pollution emission indicators). Combined with the specific research content, the second enterprise database used in this paper was China’s Polluting Enterprise Survey Database (PES). The database records in detail the energy consumption and pollutant emissions of enterprises. Among them, types of energy consumption mainly include coal, fuel oil and natural gas; the pollutant emissions mainly include sulfur dioxide, nitrogen oxides, soot, and industrial dust. The main pollutant emissions of key survey enterprises account for more than 85% of the total emissions in various regions, which is the most detailed enterprise environmental statistics database in China at present. Therefore, this database is the most suitable and comprehensive database for this study. By matching the database with the database of Chinese industrial enterprises, the final data sample of this paper was formed.

### 3.2. Index Measure

From the literature review in Section 2.1.1, we estimated the resource misallocation of enterprises based on Heish and Klenow [1]. In addition, in the pioneering paper of Heish and Klenow [1], ASIF was also used to study the problem of resource misallocation in China, so this method has strong applicability for the study of enterprise resource misallocation. The specific calculation is as follows.

It is assumed that the heterogeneous enterprise *i* in *S* industry is facing a monopolistic competitive market, and the production function of each enterprise is Cobb Douglas production function (Cobb–Douglas) with constant return to scale:

(1) $$\;Y_{si}=\;A_{si}K_{si}^{\alpha_{s}}L_{si}^{1-\alpha_{s}}$$

when an enterprise puts in factors for production, if there is no resource misallocation, the enterprise equation of profit maximization can be expressed as follows:

(2) $$\;\pi_{si}=\;P_{si}\;Y_{si}-\;WL_{si}-\;RK_{si}$$

At this time, the price of maximizing enterprise profit is:

(3) $$\;P_{si}={\left(\frac{R}{\alpha_{s}}\right)}^{\alpha_{s}}\times {\left(\frac{W}{1-\;\alpha_{s}}\right)}^{1-\;\alpha_{s}}\times \frac{1}{A_{si}}$$

when enterprises face the distortion of resource allocation, Formula (3) is no longer applicable. China’s household registration system, industrial structure and other factors limit the reasonable flow of labor to a certain extent. Similarly, the difficulty of capital acquisition is also different because of the nature, scale of enterprises and even policy orientation. In this part, by introducing the labor distortion factor $\tau_{Lsi}$ and the capital distortion factor $\;\tau_{Ksi}$ into the enterprise profit maximization equation, we measure the degree of resource misallocation of enterprises, namely:

(4) $$\;\pi_{si}=\;P_{si}\;Y_{si}-\;\left(1+\tau_{Lsi}\right)WL_{si}-\;\left(1+\;\tau_{Ksi}\right)RK_{si}$$

where $\tau_{Lsi}$ and $\tau_{Ksi}$ are the labor and capital distortions faced by enterprises. When $\tau_{Lsi}>0$, it means that the enterprise *i* faces higher labor factor costs for some reason; similarly, when $\tau_{Ksi}>0$, it means that enterprise *i* faces high financing costs or a lack of financing opportunities.

According to Melitz [15], in the monopolistic competitive market, the demand function faced by enterprises is no longer only a function of the price, but is also related to the prices of other products and the total social demand, namely:

(5) $$\;\frac{dP_{si}}{dY_{si}}=\;-\;\frac{P_{i}}{\sigma Y_{si}}$$

Formula (5) shows that when the substitution elasticity between products is σ, the demand price elasticity of products is also σ.

By finding the first-order conditions of the profit maximization equation of capital $K_{si}$ and labor $L_{si}$, we can determine the optimal price and labor–capital ratio:

(6) $$\;P_{si}=\frac{\sigma }{\sigma -\;1}\times {\left[\frac{R(1+\;\tau_{Ksi})}{\alpha_{s}}\right]}^{\alpha_{s}}\times {\left[\frac{W(1+\;\tau_{Lsi})}{1-\;\alpha_{s}}\right]}^{1-\;\alpha_{s}}\times \frac{1}{A_{si}}$$

(7) $$\;\frac{K_{si}}{L_{si}}=\frac{\alpha_{s}}{1-\;\alpha_{s}}\times \frac{W}{R}\times \frac{1+\;\tau_{Lsi}}{1+\;\tau_{Ksi}}$$

According to the first-order condition of enterprise profit maximization, the marginal revenue product of enterprise *i* can be obtained:

(8) $$\;MRPL_{si}=W\times \left(1+\tau_{Lsi}\right)$$

(9) $$\;MRPK_{si}=W\times \left(1+\tau_{Ksi}\right)$$

According to Formulas (3) and (6), the profit-maximizing price of the monopolistic competition market is higher than that of the completely competitive market, which is equivalent to the addition ($\frac{\sigma }{\sigma -1}$) of optimal price in the completely competitive market. Considering further labor misallocation and capital misallocation, according to Formulas (8) and (9), factor misallocation will be reflected in product prices. The higher $\tau_{Lsi}$ and $\tau_{Ksi}$ are, the higher the price when profit is maximized, i.e., $\tau_{Lsi}$ and $\tau_{Ksi}$ will affect labor and capital gains (MRPL and MRPK). Representing MRPL and MRPK as a dominant form, the following two formulas are available:

(10) $$\;MRPL_{si}=\;\frac{dP_{si}Y_{si}}{dL}=\;\left(1-\;\alpha_{s}\right)\times \frac{\sigma -\;1}{\sigma }\times \frac{P_{si}Y_{si}}{L_{si}}$$

(11) $$\;MRPK_{si}=\;\frac{dP_{si}Y_{si}}{dK}=\;\alpha_{s}\times \frac{\sigma -\;1}{\sigma }\times \frac{P_{si}Y_{si}}{K_{si}}$$

According to Formulas (10) and (11), the labor misallocation ($\tau_{Lsi}$) and capital misallocation ($\tau_{Ksi}$) of the *i* enterprise can be obtained:

(12) $$\;1+\;\tau_{Lsi}=\;\left(1-\;\alpha_{s}\right)\times \frac{\sigma -\;1}{\sigma }\times \frac{P_{si}Y_{si}}{WL_{si}}$$

(13) $$\;1+\;\tau_{Ksi}=\;\alpha_{s}\times \frac{\sigma -\;1}{\sigma }\times \frac{P_{si}Y_{si}}{RK_{si}}$$

Formulas (12) and (13) represent the level of capital misallocation and labor misallocation of an enterprise. Drawing on the research of Oberfield [28], the weight of capital misallocation and labor misallocation is used to measure the overall resource misallocation (mis):

(14) $$\;mis=\;\alpha_{s}\times \tau_{Ksi}+\;\left(1-\;\alpha_{s}\right)\times \tau_{Lsi}$$

Regarding the calibration of parameters, this paper draws on the practices in the pioneer work of Heish and Klenow [1], assuming that the capital use cost R is 0.1, the substitution elasticity is set to 3, and the labor share is 0.33 (1/ 3). The data of other enterprises in the process of calculation are all from the China Industrial Enterprises Database.

To test Hypotheses 1 and 2, we need to obtain the overall level of enterprise emissions and the total energy input. PES provides the amount of different pollutants discharged by enterprises and different types of energy input, so it needs to be converted by a unified standard.

In order to obtain the total amount of pollution emissions (see Appendix B.1), it is necessary to build indicators to sum up the pollutants of enterprises. Considering the differences in economic impact and the dimensions of different pollutants, this paper carries out the dimensionless treatment of different pollutants in enterprises according to China’s “Standard Management Measures for the Collection of Sewage Charges” (SMCS). The specific formula is as follows:

(15) $$\;AP_{e}=\;Q_{e}/W_{e}$$

where $AP_{e}$ indicates the number of pollution equivalents (see Appendix B.2). $Q_{e}$ indicates pollution emissions, and $W_{e}$ indicates the equivalent value of pollution (see Appendix B.3). SMCS stipulates the equivalent values of different pollutants. *e* represents different pollutants. Combining specific research content and the availability of existing data, this paper selects exhaust pollutants to represent the environmental performance of the enterprise.

Considering the different types and forms of the energy input of enterprises, this paper converts the energy input of enterprises into standard coal according to the conversion coefficient of standard coal provided by “General Principles for Calculation of Total Production Energy Consumption” of the People’s Republic of China.

## 4. Model Setting and Hypothesis Testing

### 4.1. Model Setting

Through the construction of an empirical model, this subsection discusses the environmental performance of enterprises from the perspective of resource misallocation, and verifies the four hypotheses in the second subsection. The econometric model is as follows:

(16) $$\ln tap_{it}=\theta +\alpha \;mis_{it}+\beta X_{it}+\lambda_{i}+\lambda_{t}+\varepsilon_{it}$$

where the subscript *i* represents enterprise, and the subscript *t* represents the year. $lntap_{it}$ represents the total pollution emission level of the enterprise in logarithmic form. $mis_{it}$ represents resource misallocation, which is calculated according to Hsieh and Klenow [1]. $X_{it}$ represents control variables. $\lambda_{i}$ and $\lambda_{t}$ represent the individual-fixed effect and time-fixed effect, respectively, $\epsilon_{it}$ represents the error term. The control variables selected in this paper are: (1) The level of enterprise scale (lnfixasset), which is expressed by the total fixed assets of the enterprise. According to the environmental Kuznets (EKC) hypothesis, enterprise size may have an impact on enterprise output and pollution emissions; (2) The enterprise age (age), which is expressed by the year minus the year of establishment and adding one. According to the life cycle theory, the development of enterprises will generally go through stages of growth, maturity and decline, and different stages may produce different levels of pollution emissions; (3) Enterprise financing constraint (finacons), which is expressed by the ratio of enterprise interest expenditure to fixed assets, reflecting the cost of obtaining financing; (4) The asset-liability ratio (alr), which is measured by the ratio of total liabilities to total assets. The asset-liability ratio reflects the ability of the enterprise to carry out business activities with the funds provided by creditors, which may affect the pollution emission level of the enterprise; (5) The enterprise profit level (profitlv), which is measured by the ratio of the total profit to sales revenue. To a certain extent, the profitability can reflect the ability of the enterprise to carry out green technology innovation. The results of the descriptive statistical analysis are shown in Table 1.

### 4.2. Testing Research Hypotheses

This subsection uses the empirical model in Section 4.1 to test the four research hypotheses and displays the test results.

#### 4.2.1. Testing Hypothesis 1

Table 2 shows the regression results of resource misallocation on the total pollution emission of enterprises. Column (1) is the regression result without adding any control variables. From the coefficient of resource misallocation (mis), it can be seen that resource misallocation can significantly increase the pollution emissions of enterprises. Columns (2)–(6) are the result of adding enterprise-level control variables in turn, which shows that the resource misallocation can significantly increase enterprise pollution emissions.

The regression results in Table 2 show that the resource misallocation can significantly increase the total pollution emission of enterprises. Is there a difference among different pollutants? Table 3 examines the impact of resource misallocation on the emission level of sulfur dioxide, nitrogen oxides, soot and industrial dust. Column (1) is the effect of resource misallocation on sulfur dioxide emissions. The results show that resource misallocation significantly increases sulfur dioxide emissions. Columns (2)–(4) show that resource misallocation significantly increases the emission of nitrogen oxides, soot and industrial dust, respectively. By comparing the coefficients of resource misallocation (mis), it can be seen that resource misallocation has the greatest impact on sulfur dioxide emissions, followed by industrial dust, which has the smallest impact on soot emissions. The results in Table 3 show that resource misallocation cannot only significantly increase the overall level of pollution emission, but also significantly increase the emission of individual pollutants. This largely proves the robustness of the results in Table 2.

The results of Table 2 and Table 3 show that resource misallocation can significantly increase the pollution emissions of enterprises, regardless of individual pollutant emissions or total pollution emissions. Therefore, Hypothesis 1 can be verified. In other words, resource misallocation will lead to an increase in pollutant discharge and the deterioration of environmental performance. However, the following question remains: how does resource misallocation affect the environmental performance?

#### 4.2.2. Testing Hypothesis 2

Energy consumption is closely related to the economic and environmental performance of enterprises. The problems of zombie enterprises, the loss of scale efficiency, the allocation of less production resources by high-efficiency enterprises and the excessive occupation of production resources by low-efficiency enterprises are all manifestations of resource misallocation. Compared with the state where there is no loss of resource allocation efficiency, these situations will lead to inefficiency in the total use of resources. While the inefficiency of energy consumption leads to the waste of scarce resources, it also leads to the increase in unexpected output during the production process. The final result is the double loss of enterprise environmental performance and economic performance. This is also the core content of Hypothesis 2, that is, resource misallocation leads to an increase in the average level of energy input, which in turn increases pollution emissions.

This subsection uses the model in Section 4.1 to test Hypothesis 2. Table 4 shows the empirical test results of Hypothesis 2. In Table 4, the explained variable (lntce) is the logarithm of standard coal consumed by enterprises. According to the conversion coefficient of standard coal provided by the “General Principles for Calculation of Total Production Energy Consumption” of the People’s Republic of China, this paper converts different forms of energy input into standard coal (tce), and the unit is ton. Column (1) is the regression result without adding any control variables. In order to control the possible influence of other factors, we successively add control variables in columns (2)–(6). The results from columns (1)–(6) all indicate that resource misallocation can significantly increase the energy input of enterprises. The negative externalities caused by the increase in energy input will inevitably lead to the increase in pollution emissions and the loss of environmental quality. Thus, the second hypothesis of this paper can be verified.

#### 4.2.3. Testing Hypothesis 3

According to Hypothesis 3, resource misallocation will lead enterprises to deviate from the optimal factor input ratio. Table 5 is the regression result of using the empirical model in Section 4.1 to test how resource misallocation affects the proportion of enterprise factor input. In Table 5, the variable of laben represents the labor–energy ratio, and the variable of capen represents the capital–energy ratio of enterprise. The results in columns (1)–(2) show that resource misallocation significantly reduces the labor–energy ratio, and the results in columns (3)–(4) show that resource misallocation significantly reduces the capital–energy ratio. The results in Table 5 show that the resource misallocation leads to the factor input structure in the production more inclined to the use of energy, which is not conducive to the improvement of environmental quality. Since capital investment is more sensitive to energy consumption in the production process, the absolute value of the regression coefficient in column (4) is much greater than column (2). The regression results in Table 5 fully prove Hypothesis 3. At the same time, the empirical results in Table 5 further support Hypothesis 2. Resource misallocation leads to the inefficiency of total energy input.

#### 4.2.4. Testing Hypothesis 4

The improvement of energy efficiency is an important way to mitigate the threats posed by environmental and climate change. According to Hypothesis 4, China’s energy efficiency level is still lower than the world average, and resource misallocation is not conducive to the improvement of energy efficiency. This subsection uses the empirical model in Section 4.1 to test Hypothesis 4. Energy efficiency (ee) is expressed as the amount of energy consumed per unit of GDP, that is, the energy consumption intensity. The higher the value, the lower the energy efficiency. Column (1) in Table 6 is the regression result without adding any control variables. In order to control other factors that may affect energy efficiency, we successively add control variables in columns (2)–(6). According to the results in Table 6, resource misallocation significantly increases energy consumption intensity, that is, the low efficiency of resource allocation is not conducive to the improvement of China’s energy efficiency. This also provides us with important enlightenment. In the process of environmental protection, we should pay attention to the negative impact of resource misallocation on the environment. China and other developing countries should strive to improve their efficiency in terms of resource allocation.

### 4.3. Robustness Checks

This subsection examines the possible endogenous problems between resource misallocation and environmental pollution. The process of resource misallocation is accompanied by more pollution emissions, and the deterioration of environmental quality will affect public health and the cost of emission reduction, which will negatively affect the output of enterprises. The enterprises pursuing profit maximization may further rely on the input of resources to avoid these negative effects, which aggravate resource misallocation. The possible two-way causal relationship between resource misallocation and environmental pollution will lead to the deviation of the estimation coefficient. In addition, the deviation of missing variables due to local time-varying conditions can also lead to the deviation of resource misallocation on environmental pollution. We use the lagged terms of resource misallocation as instrumental variable to solve the possible two-way causality and missing variables in model estimation. The estimated result of the instrumental variable method is shown in column (1) of Table 7. Compared with the benchmark results, the absolute value of the resource misallocation coefficient increases significantly when the instrumental variable method is used. If the possible endogenous problems are ignored, the impact of resource misallocation on environmental pollution will be significantly underestimated.

The existence of the measurement error and extreme value will also make the estimation result biased. In order to avoid the influence of measurement error and extreme value, we winsorized the top and bottom 1% of the data based on the variable resource misallocation index (mis). Column (2) is the result of the tailing treatment, where the variable of mis_w is the resource misallocation index after tailing. The regression results show that the resource misallocation still significantly increases the pollution emissions of enterprises, but compared with the benchmark results, the coefficient of resource misallocation has little difference, indicating that the benchmark result is hardly affected by measurement errors and extreme values.

## 5. Heterogeneity Analysis

The scale of the enterprise, the nature of the enterprise’s property right, and the type of enterprise may be heterogeneous in the impact of resource misallocation on environmental performance. This section further examines the impact of resource misallocation on heterogeneous enterprise environmental performance.

### 5.1. The Heterogeneity of Enterprise Scale

There is more capital and energy investment in the production process of large-scale enterprises. Under the background of the promotion championship of Chinese officials and the regional catching up strategy, the government may ignore the quality of regional economic development and carry out transitional investment or repeated construction for the purpose of local GDP growth. Therefore, compared with small-scale enterprises, the resource misallocation of large-scale enterprises may have worse environmental performance. This paper divides enterprises into large-scale enterprises, medium-scale enterprises, and small-scale enterprises according to the “Standards for the Classification of Large, Medium, and Small Industrial Enterprises”. Columns (1)–(3) in Table 8 are the regression results of enterprise scale heterogeneity. The results show that resource misallocation has a greater impact on the environmental performance of large-scale enterprises, that is, compared with small enterprises, resource misallocation leads to more pollution from large-scale enterprises.

### 5.2. The Heterogeneity of Property Rights

Columns (4)–(6) of Table 8 show the results of heterogeneity analysis of enterprise property right. As for the heterogeneity of the nature of enterprise ownership, this paper examines the state-owned enterprises, private enterprises and foreign-funded enterprises by sub-sample regression.

In China, there is a closed relationship between state-owned enterprises and the government. Firstly, the government will intervene in the financial system through administrative power, so that state-owned enterprises can obtain financial resources at lower financing costs, resulting in capital misallocation between enterprises of different ownership. The research shows that during the period 2011–2017, the real loan interest rate of Chinese state-owned enterprises was 1.6%, while the loan interest rate of private enterprises was 5.4% [29]. Capital misallocation will directly lead to overcapacity in economic operation, while emerging industries such as clean new energy and high technology will not be supported [16], which will lead to overall environmental inefficiency. Secondly, the state-owned enterprises will also aggravate labor misallocation in China, resulting in difficulties in industrial restructuring and green technology innovation. All these are not conducive to the improvement of environmental quality. Thirdly, when the economic benefits of state-owned enterprises are low, they often use their own property rights and scale advantages to seek help from the government. The government often protects state-owned enterprises due to local financial and employment pressures, interfering with the reasonable entry and exit of enterprises. Finally, due to the special nature of their ownership, state-owned enterprises bear multiple economic goals, rather than taking profit maximization as the decision-making condition, which may result in a lower resource utilization efficiency than in other enterprise ownership-types. In addition, due to the state-owned nature of energy, the government will tend to allocate more energy to state-owned enterprises, rather than to high-efficiency enterprises. Resource misallocation may further exacerbate the inefficiency of the energy input of state-owned enterprises. Table 8 of columns (4)–(6) is the result of heterogeneity regression of the nature of ownership. According to columns (4)–(6) in Table 8, the resource misallocation has a greater impact on the environmental performance of state-owned enterprises, followed by private enterprises. For foreign-funded enterprises, the environmental impact of resource misallocation is minimal, perhaps because foreign-funded enterprises have more advanced management concepts and experience, and pay more attention to the impact of resource allocation on enterprise performance.

### 5.3. The Heterogeneity of Enterprise Types

In production activities, labor factors are generally classified as cleaning factors. According to the classification of enterprise types by ASIF, light industry mainly refers to industries that provide consumer goods and hand tools, and the labor factor input is more intensive. In this case, the resource misallocation may have less of an impact on the enterprise’s pollution discharge. Heavy industry mainly refers to the industries that provide the main means of production for various sectors of the national economy, such as raw material industry, processing industry, etc., which have a large demand for capital elements and energy elements. In this case, resource misallocation may have a greater impact on the environmental performance of enterprises. Yang et al. [30] find that the correction of the distortion of factor market distortion in China’s heavy industry sector leads to the improvement of resource allocation, which reduces the energy input of the heavy industry sector by 10.57%. Columns (1)–(2) in Table 9 are the results of enterprise type heterogeneity analysis. The empirical results show that, compared with light industrial enterprises, resource misallocation have a greater impact on the environmental performance of heavy industrial enterprises.

The difference of capital intensity may also lead to a different impact of resource misallocation on environmental performance. Enterprises with high capital intensity generally mean higher energy input. In this case, the environmental impact of resource misallocation may be greater. Capital intensive enterprises mainly refer to enterprises with high capital organic composition in the production process; labor intensive enterprises refer to enterprises that need to invest a large amount of labor in the production process. Referring to Zhang et al. [31], according to the double-digit industry code of the enterprise, the enterprise is divided into capital intensive and labor-intensive industries (See Appendix C). Columns (3)–(4) in Table 9 are the analysis results of different capital intensity. The empirical results show that, compared with labor-intensive enterprises, resource misallocation have a greater impact on the environmental performance of capital-intensive enterprises. Resource misallocation will lead to more pollution from capital intensive enterprises.

## 6. Conclusions

Existing studies have mostly focused on the economic impact of resource misallocation, and believe that resource misallocation will bring about economic efficiency loss in the long run, but how the process of misallocation affects environmental quality has received little attention. Based on enterprise data, this paper examined the environmental performance of enterprises from the perspective of resource misallocation for the first time. The results are that resource misallocation can significantly increase the total pollution discharge of enterprises, a conclusion which is still valid when it comes to the discharge of a single pollutant. Further examining the potential mechanism of resource misallocation affecting enterprise environmental performance, we find that resource misallocation mainly leads to the inefficiency of the total energy input, the decrease in energy-to-capital ratio and energy-to-labor ratio, and the loss of energy efficiency, which in turn affects enterprise environmental performance. The heterogeneity analysis shows that the resource misallocation has a greater impact on the environmental performance of large-scale enterprises, state-owned enterprises and heavy-industry enterprises. Based on the above conclusions, the paper has the following policy implications.

Firstly, the allocation of resources should effectively play the role of the market, solve and improve the factors that may lead to resource misallocation, such as government intervention, policy distortions and unfair resource allocation caused by enterprise differences, and promote the improvement of environmental quality and sustainable economic development. Secondly, the concept of economic development must change and we must realize the transformation from focusing on the speed of economic growth to the unity of speed and quality benefits. In the long run, the development concept of only focusing on the speed of economic growth will produce the inefficient duplication of local construction, deepen the government’s protection of local resources and enterprises, exacerbate resource misallocation, and eventually cause double losses in terms of both economy and environment.

This paper focused on China’s environmental issues from the perspective of resource allocation, and holds that resource misallocation is an important factor restricting environmental improvement and economic development. Resource misallocation is not just a problem facing China [32], but also for many developing countries, such as India [1] and Ukraine [16,17]. The sustainable development of these countries must also pay attention to the loss of environmental quality and economic efficiency caused by resource allocation. As the largest developing country in the world, paying attention to China’s environmental issues from the perspective of resource misallocation has important significance for the environmental governance of other developing countries. However, there are differences in the economic development stage, resource endowment, industrial structure, population education level and other aspects of different countries. These are other developing countries that need to focus on solving environmental problems from the perspective of resource allocation. Of course, they will also become the focus of our future research.

## Figures and Tables

**Table 1:** Descriptive statistical analysis.

Varibles	Mean	Sd	Min	Max	N
lnfixasset	9.75	1.57	6.45	14.12	156,468
age	17.88	14.07	1.00	63	156,468
finacons	0.03	0.04	−0.00	0.22	156,468
alr	0.63	0.28	0.05	1.52	156,468
profitlv	0.02	0.09	−0.40	0.28	156,466
lnso2	3.04	1.96	−2.30	7.71	155,509
lnnox	2.39	1.97	−2.50	7.30	61,065
lnsmog	2.08	2.10	−3.41	7.09	140,903
lndust	4.18	2.74	−3.32	8.41	37,237

Notes: This table reports the summary statistics of the main variables from 2000 to 2008; where *so2*, *nox*, *smog*, and *dust* represent the emissions of industrial sulfur dioxide, industrial nitrogen oxides, industrial soot and industrial dust, respectively.

**Table 2:** Empirical results of Hypothesis 1.

Variables	lntap					
	(1)	(2)	(3)	(4)	(5)	(6)
mis	0.0051 ***	0.0082 ***	0.0082 ***	0.0082 ***	0.0082 ***	0.0082 ***
	(0.0007)	(0.0007)	(0.0007)	(0.0007)	(0.0007)	(0.0007)
lnfixasset		0.1570 ***	0.1551 ***	0.1562 ***	0.1548 ***	0.1548 ***
		(0.0060)	(0.0060)	(0.0060)	(0.0061)	(0.0061)
lnage			0.0525 ***	0.0524 ***	0.0525 ***	0.0525 ***
			(0.0063)	(0.0063)	(0.0063)	(0.0063)
finacons				0.0431 ***	0.0432 ***	0.0432 ***
				(0.0160)	(0.0160)	(0.0160)
alr					−0.0321 **	−0.0320 **
					(0.0153)	(0.0153)
profitlv						0.0013
						(0.0014)
Constant	10.4204 ***	8.8906 ***	8.7990 ***	8.7872 ***	8.8203 ***	8.8206 ***
	(0.0104)	(0.0596)	(0.0606)	(0.0608)	(0.0628)	(0.0628)
Observations	156,468	156,468	156,462	156,462	156,462	156,450
Enterprise FE	Yes	Yes	Yes	Yes	Yes	Yes
Year FE	Yes	Yes	Yes	Yes	Yes	Yes

Notes: The enterprise’s pollution emission (tap) is derived from the conversion of industrial sulfur dioxide (ton), nitrogen oxides (ton), soot (ton), and industrial dust (ton) through pollution equivalent values. Standard errors in parentheses. **, *** indicate statistical significance at 5%, 1% respectively.

**Table 3:** Effects of resource misallocation on different pollutant emissions.

Variables	(1)	(2)	(3)	(4)
	lnso2	lnnox	lnsmog	lndust
mis	0.009 ***	0.0066 ***	0.0051 ***	0.0080 ***
	(0.0008)	(0.0013)	(0.0009)	(0.0023)
lnfixasset	0.1664 ***	0.0753 ***	0.1347 ***	0.0797 ***
	(0.0068)	(0.0133)	(0.0083)	(0.0178)
lnage	0.0411 ***	0.0451 ***	0.0560 ***	0.0265
	(0.0071)	(0.0169)	(0.0087)	(0.0174)
finacons	−0.0147	0.2629 **	0.0918 ***	0.0097
	(0.0178)	(0.1181)	(0.0209)	(0.0244)
alr	−0.0525 ***	0.0518	−0.0527 **	−0.0627
	(0.0170)	(0.0322)	(0.0210)	(0.0422)
profitlv	0.0013	0.0003	0.0013	0.1731 **
	(0.0016)	(0.0015)	(0.0018)	(0.0854)
Constant	1.3476 ***	1.4143 ***	0.7052 ***	3.5224 ***
	(0.0701)	(0.1415)	(0.0860)	(0.1894)
Observations	155,491	61,056	140,889	37,232
Enterprise FE	Yes	Yes	Yes	Yes
Year FE	Yes	Yes	Yes	Yes

Notes: Standard errors in parentheses. **, *** indicate statistical significance at 5%, 1% respectively.

**Table 4:** Empirical results of Hypothesis 2.

Variables	lntce					
	(1)	(2)	(3)	(4)	(5)	(6)
mis	0.0057 ***	0.0091 ***	0.0091 ***	0.0091 ***	0.0091 ***	0.0091 ***
	(0.0005)	(0.0005)	(0.0005)	(0.0005)	(0.0005)	(0.0005)
lnfixasset		0.1752 ***	0.1737 ***	0.1747 ***	0.1740 ***	0.1740 ***
		(0.0044)	(0.0044)	(0.0045)	(0.0045)	(0.0045)
lnage			0.0414 ***	0.0413 ***	0.0414 ***	0.0414 ***
			(0.0047)	(0.0047)	(0.0047)	(0.0047)
finacons				0.0402 ***	0.0403 ***	0.0403 ***
				(0.0118)	(0.0118)	(0.0118)
alr					−0.0182	−0.0181
					(0.0113)	(0.0113)
profitlv						0.0013
						(0.0010)
Constant	0.4577 ***	−1.2500 ***	−1.3223 ***	−1.3333 ***	−1.3146 ***	−1.3145 ***
	(0.0077)	(0.0439)	(0.0447)	(0.0448)	(0.0463)	(0.0463)
Observations	156,468	156,468	156,462	156,462	156,462	156,450
Enterprise FE	Yes	Yes	Yes	Yes	Yes	Yes
Year FE	Yes	Yes	Yes	Yes	Yes	Yes

Notes: Energy input (tce) represents standard coal, which is obtained by converting different energy inputs and then summing them up. Standard errors in parentheses. *** indicate statistical significance at 1%.

**Table 5:** Empirical results of Hypothesis 3.

Variables	(1)	(2)	(3)	(4)
	Laben	Laben	Capen	Capen
mis	−0.0041 ***	−0.0039 ***	−0.8140 ***	−0.4781 ***
	(0.0008)	(0.0008)	(0.0567)	(0.0572)
lnfixasset		0.0115		17.3800 ***
		(0.0073)		(0.5004)
lnage		−0.0071		−2.9050 ***
		(0.0076)		(0.5228)
finacons		0.0025		1.8111
		(0.0192)		(1.3209)
alr		0.0417 **		2.8478 **
		(0.0183)		(1.2592)
profitlv		−0.0003		−0.0387
		(0.0017)		(0.1168)
Constant	0.7865 ***	0.6628 ***	48.0244 ***	−117.0766 ***
	(0.0124)	(0.0752)	(0.8572)	(5.1715)
Observations	156,468	156,450	156,468	156,450
Enterprise FE	Yes	Yes	Yes	Yes
Year FE	Yes	Yes	Yes	Yes

Notes: laben = labour/energy; capen = capital/energy. Standard errors in parentheses. **, *** indicate statistical significance at 5%, 1% respectively.

**Table 6:** Empirical results of Hypothesis 4.

Variables	lnee					
	(1)	(2)	(3)	(4)	(5)	(6)
mis	0.0407 ***	0.0445 ***	0.0445 ***	0.0445 ***	0.0444 ***	0.0444 ***
	(0.0007)	(0.0007)	(0.0007)	(0.0007)	(0.0007)	(0.0007)
lnfixasset		0.2095 ***	0.2089 ***	0.2105 ***	0.2056 ***	0.2053 ***
		(0.0065)	(0.0065)	(0.0065)	(0.0065)	(0.0065)
lnage			0.0139 **	0.0138 **	0.0143 **	0.0143 **
			(0.0065)	(0.0065)	(0.0065)	(0.0065)
finacons				0.0499 ***	0.0500 ***	0.0500 ***
				(0.0151)	(0.0151)	(0.0151)
alr					−0.1115 ***	−0.1104 ***
					(0.0159)	(0.0159)
profitlv						0.0152 ***
						(0.0037)
Constant	−0.9844 ***	−3.0278 ***	−3.0531 ***	−3.0693 ***	−2.9515 ***	−2.9495 ***
	(0.0099)	(0.0639)	(0.0649)	(0.0651)	(0.0672)	(0.0672)
Observations	130,661	130,661	130,655	130,655	130,655	130,655
Enterprise FE	Yes	Yes	Yes	Yes	Yes	Yes
Year FE	Yes	Yes	Yes	Yes	Yes	Yes

Notes: Energy efficiency (ee) is expressed as the amount of energy consumed per unit of GDP. Standard errors in parentheses. **, *** indicate statistical significance at 5%, 1% respectively.

**Table 7:** Endogenous problems.

Variables	IV	Winsor
	lntap	lntap
mis	0.0258 ***	
	(0.0084)	
mis_w		0.0082 ***
		(0.0007)
lnfixasset	0.1664 ***	0.1548 ***
	(0.0144)	(0.0061)
lnage	0.0239 ***	0.0525 ***
	(0.0087)	(0.0063)
finacons	0.0337 **	0.0432 ***
	(0.0154)	(0.0160)
alr	−0.0332	−0.0320 **
	(0.0204)	(0.0153)
profitlv	0.0004	0.0013
	(0.0014)	(0.0014)
Constant	8.9256 ***	8.8203 ***
	(0.2189)	(0.0628)
Observations	89,952	156,450
Enterprise FE	Yes	Yes
Year FE	Yes	Yes

Notes: Standard errors in parentheses. **, *** indicate statistical significance at 5%, 1% respectively.

**Table 8:** Results of heterogeneity analysis of enterprise scale and ownership.

lntap	Enterprise Scale			Property Rights		
	(1)	(2)	(3)	(4)	(5)	(6)
	Large Scale	Medium Scale	Small Scale	State Owned	Private	Foreign
mis	0.0078 ***	0.0082 ***	0.0026	0.0144 ***	0.0077 ***	0.0069 ***
	(0.0008)	(0.0016)	(0.0034)	(0.0021)	(0.0009)	(0.0021)
lnfixasset	0.1161 ***	0.1507 ***	0.1090 ***	0.0935 ***	0.1478 ***	0.1787 ***
	(0.0076)	(0.0150)	(0.0304)	(0.0165)	(0.0085)	(0.0218)
lnage	0.0442 ***	0.0205	−0.0010	0.0471 ***	0.0360 ***	0.0925 ***
	(0.0084)	(0.0128)	(0.0234)	(0.0148)	(0.0095)	(0.0335)
finacons	0.1037	0.0349 **	0.0831	0.0225	0.0391 **	0.4060
	(0.0763)	(0.0164)	(0.0964)	(0.0950)	(0.0156)	(0.2949)
alr	−0.0014	−0.1251 ***	−0.0743	−0.1356 ***	0.0349	0.0350
	(0.0186)	(0.0347)	(0.0835)	(0.0298)	(0.0242)	(0.0525)
profitlv	0.0008	0.0024	0.0051	0.0045	0.3056 ***	0.0004
	(0.0015)	(0.0052)	(0.0068)	(0.0044)	(0.0575)	(0.0016)
Constant	8.9579 ***	9.2685 ***	10.2830 ***	9.8613 ***	8.9162 ***	7.4610 ***
	(0.0738)	(0.1684)	(0.3812)	(0.1858)	(0.0843)	(0.2360)
Observations	102,635	41,733	12,082	33,190	77,975	19,783
Enterprise FE	Yes	Yes	Yes	Yes	Yes	Yes
Year FE	Yes	Yes	Yes	Yes	Yes	Yes

Notes: Standard errors in parentheses. **, *** indicate statistical significance at 5%, 1% respectively.

**Table 9:** Heterogeneity analysis of enterprise types.

lntap	Enterprise Types			
	(1)	(2)	(3)	(4)
	Light Industry	Heavy Industry	Capital Industry	Labour Industry
mis	0.0069 ***	0.0097 ***	0.0100 ***	0.0068 ***
	(0.0010)	(0.0010)	(0.0010)	(0.0010)
lnfixasset	0.1555 ***	0.1474 ***	0.1486 ***	0.1645 ***
	(0.0088)	(0.0086)	(0.0082)	(0.0091)
lnage	0.0490 ***	0.0509 ***	0.0497 ***	0.0508 ***
	(0.0095)	(0.0087)	(0.0084)	(0.0098)
finacons	0.1971 ***	0.0357 **	0.0331 *	0.2967 ***
	(0.0751)	(0.0169)	(0.0169)	(0.0849)
alr	−0.0264	−0.0418 **	−0.0397 *	−0.0226
	(0.0223)	(0.0213)	(0.0205)	(0.0229)
profitlv	0.0009	0.0042	0.0055	0.0006
	(0.0014)	(0.0044)	(0.0042)	(0.0014)
Constant	8.4150 ***	9.2481 ***	9.1447 ***	8.3608 ***
	(0.0899)	(0.0893)	(0.0857)	(0.0927)
Observations	73,690	82,760	91,280	65,170
Enterprise FE	Yes	Yes	Yes	Yes
Year FE	Yes	Yes	Yes	Yes

Notes: Standard errors in parentheses. *, ** and *** indicate statistical significance at 10%, 5% and 1% respectively.

## Data Availability

The data presented in this study are available on request from the corresponding author.

## Appendix A. Data Processing

This paper eliminates outlier samples through the following processes: deleting the samples of total output value, industrial sales, industrial added value, total assets and fixed assets less than or equal to zero; deleting the samples whose total assets are less than or equal to fixed assets, the number of employees is less than 10 and the subsidy income is less than zero; considering the fact that the annual industrial sales of non-state-owned enterprises in ASIF are all above CNY 5 million, in order to reduce the resulting deviation caused by heterogeneity analysis, this paper excludes all samples of non-state-owned enterprises with an annual industrial output value of less than CNY 5 million.

## Appendix B. Further Explanation of Indicator Definition

### Appendix B.1. Pollution Emissions

The pollutants in this paper mainly include industrial sulfur dioxide ($so_{2}$), nitrogen oxides (nox), soot (smog) and industrial dust (dust).

### Appendix B.2. Pollution Equivalent

Pollution equivalent refers to a comprehensive index or unit of measurement to measure the environmental pollution caused by different pollutants according to the harmful degree of pollutants or pollution discharge activities to the environment and the technical economy of treatment. The pollution degree of different pollutants with the same pollution equivalent in the same medium is basically the same.

### Appendix B.3. Pollution Equivalent Value

The pollution equivalent value is based on the harmful degree of the main pollutants in the specified unit of environmental pollution factors, the toxicity to organisms and the treatment cost, compared with other pollutants, there is a considerable value.

## Appendix C. Industry Division

**Light Industry**	**Heavy Industry**
Processing of foods (13)	Petroleum (25)
Food manufacturing (14)	Raw chemicals (26)
Beverage manufacturing (15)	Chemical fiber manufacturing (28)
Textile (17)	Rubber products industry (29)
Textile and apparel, shoes and	Plastic products industry (30)
hat manufacturing (18)	Non-metallic minerals (31)
Leather (19)	Ferrous metal smelting (32)
Timber (20)	Non-ferrous metal smelting (33)
Furniture (21)	Metal products (34)
Paper and paper products industry (22)	General machinery (35)
Printing and recording media (23)	Special machinery (36)
Articles for cultures and sports (24)	Transport equipment (37)
Medicines (27)	Electrical machinery (39)
Manufacture of artwork (42)	Communication equipment (40)
	Measuring instrument (41)
	Waste resources and waste materials
	recycling and processing (43)
